# Community health workers at the dawn of a new era: 3. Programme governance

**DOI:** 10.1186/s12961-021-00749-3

**Published:** 2021-10-12

**Authors:** Simon Lewin, Uta Lehmann, Henry B. Perry

**Affiliations:** 1 Division of Health Services, Norwegian Institute of Public Health, Oslo, Norway; 2 Health Systems Research Unit, South African Medical Research Council, Cape Town, South Africa; 3 School of Public Health, University of the Western Cape, Cape Town, South Africa; 4 Health Systems Program, Department of International Health, Johns Hopkins Bloomberg School of Public Health, Baltimore, MD United States of America

**Keywords:** Community health workers, Lay health workers, Governance, Governing, Health systems, Health workforce, Sustainable Development Goals (SDGs), Universal Health Coverage

## Abstract

**Background:**

Community health workers (CHWs) can play a critical role in primary healthcare and are seen widely as important to achieving the health-related Sustainable Development Goals (SDGs). The COVID-19 pandemic has emphasized the key role of CHWs. Improving how CHW programmes are governed is increasingly recognized as important for achieving universal access to healthcare and other health-related goals. This paper, the third in a series on “Community Health Workers at the Dawn of a New Era”, aims to raise critical questions that decision-makers need to consider for governing CHW programmes, illustrate the options for governance using examples of national CHW programmes, and set out a research agenda for understanding how CHW programmes are governed and how this can be improved.

**Methods:**

We draw from a review of the literature as well as from the knowledge and experience of those involved in the planning and management of CHW programmes.

**Results:**

Governing comprises the processes and structures through which individuals, groups, programmes, and organizations exercise rights, resolve differences, and express interests. Because CHW programmes are located between the formal health system and communities, and because they involve a wide range of stakeholders, their governance is complex. In addition, these programmes frequently fall outside of the governance structures of the formal health system or are poorly integrated with it, making governing these programmes more challenging. We discuss the following important questions that decision-makers need to consider in relation to governing CHW programmes: (1) How and where within political structures are policies made for CHW programmes? (2) Who implements decisions regarding CHW programmes and at what levels of government? (3) What laws and regulations are needed to support the programme? (4) How should the programme be adapted across different settings or groups within the country or region?

**Conclusion:**

The most appropriate and acceptable models for governing CHW programmes depend on communities, on local health systems, and on the political system in which the programme is located. Stakeholders in each setting need to consider what systems are currently in place and how they might be adapted to local needs and systems.

**Supplementary Information:**

The online version contains supplementary material available at 10.1186/s12961-021-00749-3.

Key messages box 1: summary **Key findings**

We discuss the following important questions that decision-makers need to consider in relation to governing CHW programmes:

- How and where within political structures are policies made for CHW programmes?
- Who implements decisions regarding CHW programmes, and at what levels of government?
- What laws and regulations are needed to support the programme?
- How should the programme be adapted across different settings or groups within the country or region?

**Key implications**

- Governing CHW programmes is complex because of the location of these programmes at the interface between the formal health system and communities.
- How CHW programmes are governed affects many other processes in these programmes, including management, resourcing, accountability, and ultimately performance and sustainability.
- The most appropriate and acceptable models for governing CHW programmes depend on communities, on local health systems, and on the political system in which the programme is located. Stakeholders in each setting need to consider what systems are currently in place and how they might be adapted and aligned with local needs and systems.
- The revitalization of large-scale CHW programmes in a number of contexts provides opportunities to explore how different models of governing CHW programmes impact on the quality of care delivered, their responsiveness to local needs, and their sustainability.

## Background

A key obstacle to achieving health goals is the chronic shortage and poor distribution of health workers in many settings [1–3]. Community health workers (CHWs), sometimes called lay health workers, represent one strategy for addressing these issues while simultaneously strengthening community health systems [4]. Globally, CHW is a term used to describe a very diverse group of largely community-based health workers that perform a large range of functions related to health promotion, healthcare delivery, and social development. Their scope ranges from being “frontline” providers of a range of primary healthcare (PHC) services, to single intervention providers (for example, providing support for people on TB treatment), health promoters, and providers whose main task is to refer people to health facilities [5]. CHW programmes operate in many settings, including in low-, middle-, and high-income countries, and these programmes vary widely across a range of factors, including their size, scope, how they are funded, how they are organized, and whether the CHWs are employed by the publicly funded health sector or through other mechanisms [6].

The awareness of the potential of CHWs to address many critical gaps in health systems across settings, together with the COVID-19 pandemic and the critical role of CHWs for the pandemic response [7], is arguably creating a new era for national CHW programmes. Thus, it is an appropriate moment to step back and examine issues that are critical to the long-term effectiveness of national CHW programmes, including how these programmes are governed. Improving how CHW programmes, and health systems more broadly, are governed is increasingly recognized as important in achieving universal access to healthcare and other health-related goals. “Good governance” has for some time been seen as a key goal in itself [8]. Nevertheless, questions of how CHW programmes should be governed remain largely unresolved [9, 10] and have received insufficient attention from researchers [11–13].

In 2014, we considered issues relevant to governing CHW programmes in a larger report entitled *Developing and Strengthening Community Health Worker Programs at Scale: A Reference Guide and Case Studies for Program Managers and Policymakers* [14, 15]. More recently, one of us (HBP) gathered together into a book the most comprehensive and current descriptions available for 29 national CHW programmes [16]. This paper, the third in the current series of articles on “Community Health Workers at the Dawn of a New Era”, builds on these earlier efforts and seeks to draw out the principles that continue to have relevance for the new era that CHW programmes are entering. We draw on the 2014 report and on descriptions of national CHW programmes mentioned above; and from the knowledge and experience of those involved in planning and managing CHW programmes. This knowledge and experience was gathered through a series of face-to-face and online meetings of those involved in writing the 2014 report, and through peer review feedback on that report. The questions for decision-makers outlined in this paper were further refined through discussion among the authors of this paper and through engagement with the literature on governing in the context of health systems.

This article, and the CHW Reference Guide chapter from which it draws [17], aim to: raise critical questions that decision-makers need to consider for governing CHW programmes, illustrate the options for governance using examples of national CHW programmes, and set out a research agenda for understanding how CHW programmes are governed and strategies for improving how they are governed.

## What is meant by “governing” in the context of health systems?

Governing is a challenging concept to define. Within the WHO health systems framework, “governance and leadership” is viewed as one of the six building blocks of a health system, and is seen as supporting the achievement of health systems goals by “ensuring strategic policy frameworks exist and are combined with effective oversight, coalition building, the provision of appropriate regulations and incentives, attention to system-design, and accountability” [18] (p. 3). This definition, while useful, does not capture adequately the processual nature of governing health systems. Governing health systems has therefore also been conceptualized in terms of inputs, processes, and outputs [19]. Governance inputs, it is suggested, comprise how and by whom the institutions governing the health system are constructed and managed. This includes “participation”—the stakeholders involved in developing health policies; and “consensus orientation”—the extent to which government officials collaborate with or involve other stakeholders in formulating goals and policies for health. Governance processes concern how administrative procedures governing the health sector are implemented. This includes transparency, accountability and control of corruption. Finally, governance outputs in this approach can be seen as the benefits that should result from the implementation of governance rules and processes within a health system. Different political systems (i.e., the different actors, structures, processes, and activities through which power is used to shape and implement policies in different settings) emphasize different governance outputs, and these include measures of how well the health system responds to population needs, equity of access to health services, and efficient use of health resources [19].

Since CHW programmes are embedded in communities as well as in health systems, in this paper we draw on a somewhat broader understanding of governance, which emphasizes “the complex mechanisms, processes and institutions through which citizens and groups articulate their interests, mediate their differences and exercise their legal rights and obligations” [20] (p. 14). As this description suggests, governing involves ongoing interactions between multiple *actors—*such as healthcare decision-makers, community representatives, and agencies—and *structures*—the laws, policies, resources, and beliefs within which these actors work [21]. Governing is therefore a continuous *process* rather than a static set of policies and structures, and is fundamentally relational. Consequently, the process of governing is closely linked to context and actors and may change over time as societies, health systems, and CHW programmes evolve. Our approach to governing in this paper is congruent with other recent conceptual thinking that takes a less “government-centred” view and focuses more on the roles of a wider range of actors, and the rules that shape their collective action to make decisions [22, 23]. Within the health sector, these actors may include citizens and citizen organizations, labour unions, nongovernmental organizations (NGOs), multilateral and bilateral organizations, and donors, sometimes working together through coalitions to influence decisions [24]. These models also highlight not only the *formal* but also the *informal* rules, “…governing the demand and supply of health care, and both the formal and informal ways in which the rules are made, changed, monitored and enforced…” [23] (p. 1343). These broader approaches are helpful when considering how CHW programmes are governed, given these programmes’ location at the boundary of formal health systems and community structures and their roles as boundary spanners in the “micro-practices” of governing [25, 26].

## Why is governing an important issue for CHW programmes?

**Table:** 

Key message box 2	
Governing comprises the processes and structures through which individuals, groups, programmes, and organizations exercise rights, resolve differences, and express interests	

How CHW programmes are governed is generally not well understood, partly because these programmes are enormously diverse and often run in parallel to or as an appendix to formal health systems, and partly because this is an under-researched area. But just as for all other parts of the health system, how CHW programmes are governed affects many other processes in these programmes, including management, resourcing, accountability, and ultimately performance and sustainability. Some of the important decision parameters in relation to governing CHW programmes include the extent to which these programmes are part of the formal health system as well as the extent to which CHWs are recognized formally as a group of health workers within the health system. Other important parameters are the extent of decentralization of authority for governing CHW programmes and for their management; the scale of the programme; and the roles which key actors, including communities and/or service users, have in shaping and directing the programmes. In addition, decisions on how CHW programmes are governed are influenced by how, and by whom, resources are obtained and administered.

Different decisions on these parameters, in response to specific contexts and needs, result in vastly different governance models for CHW programmes. For instance, in some settings, programmes are not part of the formal facility-based health system but have structures that provide good links to this system. This model is used within the accredited social health activist (ASHA) programme in India and the BRAC (Building Resources Across Communities) programme in Bangladesh [14]. In other settings, programmes are integrated into the formal health system and are well supported within it. Examples of this include the family health teams in Brazil (with their CHWs, who are called community health agents) and the health extension worker programme in Ethiopia [14]. These different models for governing CHW programmes have implications, in turn, for how programmes are financed and funded; how and by whom CHWs are selected and trained; how CHWs are supported and supervised; how CHWs are paid; how communities are involved, and many other issues. We discuss the implications of these different configurations in more detail below.

Because CHW programmes, to varying degrees, are at the interface of formal health systems and community systems [27], and can involve a wide range of stakeholders at local, national, and international levels, their governance is particularly complex and relational. Further, many programmes aspire to participatory governance, in which communities and other actors shape the structure and functions of the programmes and are involved in monitoring and accountability mechanisms [28]. However, programmes have often failed to successfully implement participatory governance, raising questions about the feasibility of the models used [28–30]. In addition, many programmes have been scaled up very quickly with little thought being given to how they should be governed [29].

Improving how CHW programmes (and other health system programmes) are governed requires addressing a range of enabling factors. These include, for example, clarifying the goals for the CHW programme; creating stronger standards regarding the selection and training of CHWs; and establishing more meaningful involvement of, and accountability to, the stakeholders linked to these programmes, including local communities and recipients of CHW care. Governing CHW programmes therefore requires financial and other resources, and how these resources are managed will in turn affect the extent to which good governance can be achieved ([31] p. 3; [32] p. 243). We discuss these factors in more detail below, while Additional file 1: Table S1 provides a summary of governance principles within healthcare more broadly.

## What are important considerations for governing CHW programmes?

**Table:** 

Key message box 3	
How CHW programmes are governed affects many other processes in these programmes, including management, resourcing, accountability, and ultimately performance and sustainability	

Important questions that decision-makers need to consider in relation to governing CHW programmes include:

1. How and where within political structures are policies made for CHW programmes?
2. Who implements decisions regarding CHW programmes, and at what levels of government?
3. What laws and regulations are needed to support the programme?
4. How should the programme be adapted across different settings or groups within the country or region?

We discuss each of these questions below, drawing on examples from case studies of CHW programmes in Brazil, Ethiopia, India, Pakistan, and South Africa. Table 1 summarizes the sub-questions for each of the main questions, and these are also discussed further in the CHW Reference Guide chapter from which this paper is drawn [17]. Further detail on the CHW programmes used as examples is provided in Additional file 1: Tables S2 and S3, which provide a cross-country comparison of issues in the governing of these CHW programmes and policies that affect individual CHWs. These tables also include additional material that complements the issues raised in the paper.

**Table 1:** Governing community health worker programmes: important questions and sub-questions (based on [17])

Questions	Sub-questions
How and where within political structures are policies made for CHW programmes?	*Where are policy decisions for CHW programmes made and by whom?* Where are laws and regulations relevant to health initiated? Do laws need to be initiated by the cabinet or by the parliament? Can other stakeholders initiate laws or regulations through other mechanisms? Who can initiate such laws and regulations for CHW programmes? Do laws need to be initiated by a government minister or a ministerial permanent secretary? Who are the key stakeholders for policies related to community health services? To what extent are these key stakeholders consulted and involved in policy-making for community health services? Are there important groups who are not consulted or involved, for example, on the basis of ethnicity, gender, or sexual orientation? To what extent is there a consensus orientation in which government authorities cooperate with other stakeholders in policy development? How are inputs solicited from stakeholders? Do the approaches used foster the participation of all key stakeholders? How are the varied objectives, motivations, and views of different stakeholders reconciled within the policy process? *How might important historical legacies shape CHW-related policy-making?* Are there important health system legacies in relation to governance, finance or service delivery arrangements^a^ that may shape CHW-related policy-making? Are there important political system legacies in relation to institutions, interests or ideas^b^ that may shape CHW-related policy-making? To what extent are these historical legacies in alignment with the planned policy? What scope is there for reshaping the policy or bypassing these legacies? *How might wider health and political systems goals in a particular context influence how CHW programmes are governed?* What goals are emphasized currently within the health and political system in a particular context? To what extent will CHW-related policies help to achieve these goals, and how can this be demonstrated within the policy process? What changes need to be made to proposed CHW policies to better align them with relevant governance goals? Where CHW-related policies diverge from prioritized governance goals, how can this be justified and advocated for within the policy process? Are there persons with political influence who can advocate for CHW programmes?
Who implements decisions regarding CHW programmes, and at what levels of government?	What factors might affect the successful implementation of the policy? In what ways can potential barriers be overcome or minimized and facilitators harnessed? Is there a clear plan for implementation of policy decisions that includes the objectives to be achieved, adequate resources, and a time frame, and that addresses important barriers and facilitators? How will implementation ensure that key governance goals, such as equity, participation, and accountability, are maximized? How will implementation of policies be monitored and evaluated to ensure that their objectives are met?
What laws and regulations are needed to support the programme?	Which laws and regulations are relevant to the governing and scale-up of CHW programmes? How are these laws and regulations translated into rules and procedures that may affect programme implementation in the field, and who has responsibility for this? Will any changes be required to these laws and regulations to allow the programme to be scaled up as intended? Will any new laws and regulations be needed? Where laws or regulations need to be promulgated or amended, which government bodies would be responsible for leading this process? Which other bodies would need to be involved in this process? Are there key laws or regulations that may act as critical barriers or bottlenecks to policy implementation and that should therefore be priorities for promulgation or amendment? What is the likely time frame for these legislative or regulatory processes? Can scale-up be implemented in parallel to changes in laws and regulations?
How should the programme be adapted across different settings or groups within the country or region?	Is the programme targeted towards specific groups or settings in the country or region? Are there important differences across groups or settings in the country or region that may affect roll-out of the programme and that may require its adaptation? This could include differences in relation to sociodemographic factors such as age, gender, sex, sexual orientation, ethnicity, religious affiliation, migration background, and language, as well as socioeconomic factors such as income and education or literacy levels How will the programme be adapted, if this is needed?

^a^Governance arrangements are concerned with political, economic, and administrative authority in the management of health systems, as noted above. Financial arrangements include funding and incentive systems, while delivery arrangements include human resources for health, as well as service delivery [61]

^b^Drawing on political science theory, the term “institutions” is used here to refer to both the formal and informal structures and processes of policy-making (constitutional rules, structures through which decision are made, and features of the policy process, such as the level of transparency). The term “interests” concerns the stakeholders who shape a policy and their views on whether the policy will have benefits or drawbacks for them or others. The term “ideas” refers to the values and knowledge held by stakeholders, including those in government and civil society, and comprises information from both research and experience [62, 63]

## How and where within political structures are policies made for CHW programmes?

CHW programmes experience a range of challenges in relation to policy processes. In particular, CHW programmes typically straddle health and community systems [27, 33] and may also be tacked on to other programmes or the formal health system more broadly. This may lead to these programmes not being fully integrated into the formal health system or being seen as peripheral to it. Policies to govern these programmes may consequently be lacking or may not be “fit for purpose”, and it is therefore important to consider how and where policies for CHW programmes are made. In addition, wider policy decisions, such as efforts to decentralize or centralize decision-making for health services, may have important impacts on CHW programmes [34, 35]. These policy decisions (another example being whether to develop a volunteer-based or fully remunerated CHW programme) need to be distinguished from implementation decisions (such as the timetable for continuing education of CHWs within a particular district). We discuss below the key issues to consider for CHW programmes in relation to how and where within political structures—the institutions, groups, laws, and regulations of the political system—policies for these programmes are made.

**Table:** 

Key message box 4	
Key issues to consider for CHW programmes in relation to policy processes include where policy decisions are made and who is involved; how important historical legacies might shape policy-making; and how wider health and political system goals may influence the governing of CHW programmes	

### Where are policy decisions made and who is involved in this?

Authority to make policy and operational decisions regarding CHW programmes is located at different levels of government within different countries, depending on the country’s administrative arrangements (for instance, the extent to which decision-making is decentralized; see Boxes 1 and 2 or historical policy legacies [see below]). In some countries, such authority may be located with the national ministry of health (MOH). In other countries, regional departments of health or legislatures may have authority to develop health policies, or such authority may have been delegated to an independent body, such as a CHW commission. Each of these scenarios has different benefits and drawbacks. For example, where policy authority is located at national level, it may be easier to achieve consistency of approach for CHW programmes across a country. However, policy-making may be very removed from the day-to-day running of CHW programmes and may therefore not be very responsive to local conditions and needs [36]. An important question in many settings is how structures within the MOH might be adjusted to improve the governance of CHW programmes? MOHs in many countries are highly fragmented, with vertical disease programmes that receive external support operating with relative independence, thereby impeding collaboration on integrated programming. Further, there are relatively few MOHs that have a directorate responsible specifically for primary healthcare, and where such directorates exist, they are often relatively weak. Given this context, should MOHs establish a separate directorate with responsibility for CHW programme strategy and funding [37] or would other types of structures for governing CHW programmes be more effective? Boxes 1 and 2 describe examples of how the locus of decision-making can impact on CHW programmes.

It is also important to consider the range of stakeholders that may have roles in designing CHW policies. The extent to which there is wide participation in this process depends on the orientation of the political system within a particular context; the formal and informal power which stakeholders are able to exert; and the attitudes of those driving a particular policy process. Which stakeholders are involved in CHW policy-making, how these stakeholders are involved, and whether there are important groups who are not consulted or involved (for instance, on the basis of ethnicity, gender, or sexual orientation) have important implications for programmes. For example, where it is not clear who has final responsibility for policy-making, decisions may not be made or may be very delayed. Also, where policy decision-making is dispersed across a range of stakeholders, important inconsistencies can develop across programme policies. Additional file 1: Table S3 and the CHW Reference Guide [14] describe a range of questions that need to be considered in relation to stakeholder involvement.

Those wishing to develop or change policies governing CHW programmes need to consider where laws and regulations relevant to health are initiated and who can initiate these [20]. In addition, consideration needs to be given to what provisions there are locally for accountability and support. For example, what recourse citizens have if they feel that CHWs are not carrying out their duties adequately. This is addressed in more detail in Paper 9 in the series, which explores relationships with communities [38].

Box 1. Governance within the Brazilian Family Health Programme: where policy decisions are made [39] In Brazil, the constitution adopted in 1988 reinforced the role of state (provincial) and municipal governments in implementing public policies, while the central government has the role of issuing the main guidelines for implementing public policies. Later legal provisions have shifted more responsibility for the management and organization of health services over to municipal governments, while at the same time emphasizing the technical and financial role of the central government and the states. In this decentralized model, municipalities have the authority to decide whether to implement the Family Health Programme. Once a decision to implement is made, the local government determines the organization of the programme in their municipality, for example specifying the number of family health teams they want to establish and selecting the areas to which these teams will be assigned.

The positive effects on the programme resulting from such a process of implementation appears to be more local ownership of the implementation and improved local management of the programme. On the other hand, the process could lead to unprepared and uncommitted local management as well as heterogeneity of implementation

Box 2. Governance of programmes supported by the National Rural Health Mission in India: where policy decisions are made [39] The three tiers of governance (central government, state, and panchayats) in India pose challenges for a range of government programmes, including for carrying out certain functions of the National Rural Health Mission (an initiative of the Ministry of Health and Family Welfare to strengthen rural health services). An evaluation from 2009 reported that transfers of funds to lower levels of governance were being held up at the state levels. The evaluation proposed direct disbursement of funds from the central government to the panchayats as a solution to this problem. However, it was noted that this change may be difficult, given that health is defined as a state responsibility in the constitution of India. The evaluation suggests that individual states would like to gain more autonomy from the centre. However, states are reluctant to devolve the necessary powers to govern CHW programmes to the panchayat level where PHC centres and sub-centres are located. Similar tensions were reported between the central government and the states in relation to financing of the programme

### How might important historical legacies shape CHW-related policy-making?

CHW programmes never start from a blank slate but are shaped by historical legacies including previous and current policies, experiences, practices, and cultures. For example, a CHW programme may have been established with the specific purpose of improving equity of access to healthcare for historically marginalized groups, such as populations living in geographically remote areas [40]. The Brazilian Family Health Programme, for instance, has its antecedents in a regional programme, established to respond to a severe drought in one of the most impoverished parts of the country (see Additional file 1: Table S2, row 3) [41]. The model developed in this setting has shaped the programme across the country. Programmes may also be shaped by specific health system legacies: for instance, CHW policies may need to take into account an existing nurse auxiliary cadre or may need to absorb an existing network of community health volunteers. Efforts to establish a national CHW policy framework in South Africa, for example, have been influenced by the absence of a national CHW programme and the presence of a large number of small- to medium-sized programmes, largely managed by NGOs, in which CHWs have different scopes of practice and levels of training (see Additional file 1: Table S2, rows 2 and 4).

Historical legacies are important as they may determine stakeholders’ views of and engagements with policies. These legacies can also constrain what is possible—for instance, it may be difficult to make substantial changes to CHWs’ existing scopes of practices, such as introducing curative tasks to a programme focusing on health promotion; or to the types of recipients targeted, for example, from women and children to everyone in the household. Additional file 1: Table S3 and the CHW Reference Guide [14] describe a range of questions that need to be considered in relation to historical legacies that shape CHW-related policy-making.

### How might wider health and political system goals in a particular context influence how CHW programmes are governed?

How CHW programmes are governed will be influenced by the particular goals or benefits (sometimes called governance outputs) that have been prioritized within a specific health or political system. CHW and other health policies may be assessed by decision-makers in relation to the extent to which they help to achieve these goals or outputs. These goals may include improved equity, greater efficiency in the delivery of services, more decentralized services, or greater involvement of the private sector in the delivery of services.

There are a number of ways in which wider health and political system goals may influence how CHW programmes are governed. Firstly, it will be difficult to develop CHW programme policies and governance processes where these do not align with wider goals. For instance, developing structures to allow CHWs to work more closely with private sector providers, such as drug dispensers, may not be feasible if such arrangements are not seen as legitimate or important within the wider health system. In contrast, ways of governing CHW programmes that align closely with political system goals, such as the decentralization of services, may be easier to develop and implement.

Secondly, health and political system goals may drive the development, or indeed the demise, of a CHW programme. In many settings, programmes have been developed or scaled up to help achieve the goal of improved equity in access to health services. In Ethiopia, for example, the Health Extension Worker Programme aims to improve access to care for rural populations particularly (Additional file 1: Table S2, row 4). In South Africa, efforts by the first democratic government beginning in 1994 to improve equity and quality in PHC prioritized nurses as the lead healthcare provider and for a long time viewed CHWs as second-rate-care providers. Consequently, funding and support for CHW programmes declined, and many programmes ceased to function [40] (Additional file 1: Table S2, row 4).

Additional file 1: Table S3 and the CHW Reference Guide [14] describe a range of questions that need to be considered in relation to how wider health and political system goals in a particular context might influence how a CHW programme is governed. There are a number of ways, both formal and informal, in which these questions can be considered. Those governing CHW programmes can reflect on the goals of the programme, as well as those of the wider health and political system, and the extent to which CHW policies will help to achieve these wider goals. Wider consultations, such as deliberative dialogue processes, may be useful in identifying current and future health and political system goals, in considering how CHW policies align with these, and in assessing how the governing of CHW programmes needs to shift in order to support important health and political system goals. A number of policy analysis tools are available that could be useful in this process [42–45].

## Who implements decisions regarding CHW programmes, and at what levels of government?

After a policy decision has been made, the next key challenge is transforming this policy into practical actions. Policy implementation is challenging in most settings for a range of reasons, including the complexity of both the health system and communities, their interactions, the multiplicity and diversity of actors involved, and the complexities of maximizing participation and reducing inequities. Furthermore, there are a number of contextual factors to consider, including (1) identifying, allocating, and disbursing limited financial resources, (2) deficits of other resources, including human resources for healthcare delivery and management, (3) competing priorities within and beyond the health system, and (4) challenging physical environments, such as very remote communities. A key challenge for governing CHW programmes is to align and catalyse these factors. Where such alignment does not occur, implementation is likely to take place in an unsystematic way or be slowed by a range of obstacles (Box 3). Careful and systematic planning is needed to ensure that CHW programme policies are implemented as intended. Additional file 1: Table S3 and the CHW Reference Guide [14] describe a range of questions (adapted from [20, 46]) that need to be considered by policy-makers when planning the implementation of policies for CHW programmes.

Box 3. Involving communities in CHW programme implementation in Zimbabwe (from [39]) Studies analysing the implementation of the village health worker (VHW) programme in Zimbabwe provide an in-depth analysis of why such local citizen bodies may have failed to stimulate meaningful community involvement. These studies suggest that the government, while attempting to redirect resources to the village level, developed an increasingly large bureaucracy that reinforced centralization of power, and local citizen bodies became extensions of the central government structures. People’s representation was supposed to be mediated through village and district committees. However, these structures were regarded by communities as remote, and as a part of civil service structures that were accountable to the government and not to poor people within communities. Effective popular mobilization in the planning and development of the VHW programme was seen to have declined inversely in relation to the bureaucratization of the programme

## What laws and regulations are needed to support the programme?

The governing and implementation of CHW programmes may be shaped or constrained by existing laws or regulations. As noted above, these “policy legacies” [47] may include regulations regarding the kinds of healthcare providers who can prescribe and dispense different types of medications or undertake specific tasks, such as giving vaccinations. These legacies may also include laws regarding the disbursement of funds from health departments to community structures who may be responsible for supporting CHWs. CHW programmes may experience challenges if laws and regulations that are needed to enable effective programme functioning are not put in place in a timely manner or if existing laws and regulations are not amended as needed. For example, regulations in Brazil regarding the need to advertise civil service posts nationally were changed to help ensure that CHWs employed by the Family Health Programme came from the community in which they were to work [48]. In South Africa, it has been argued the functioning of CHW programmes was hampered by poor regulation that limited the rights of CHWs and contributed to low pay levels [49].

Alignment of legal and regulatory frameworks is therefore needed for large-scale programmes to function effectively [50]. These need to address issues related to CHWs, such as selection and remuneration, as well as how CHW programmes interface with the wider health system, such as governance structures for PHC. Those developing and scaling up CHW programmes therefore need to consider which existing laws and regulations need to be taken into account, and whether changes to these are needed to ensure the effective governing of the programme and its implementation as intended. Additional file 1: Table S3 and the CHW Reference Guide [14] describe a range of questions that need to be considered in relation to laws and regulations.

## How should the programme be adapted across different settings or groups within the country or region?

For CHW programmes operating at scale, there may be a tension between, on the one hand, adopting a fairly standard approach to the governing of programmes and to their implementation and, on the other hand, trying to ensure that the programme is tailored to the needs of different settings or groups. The latter approach, while more resource-intensive and more difficult to implement, may help to ensure that the programme is embedded in and tailored to local communities and health services. The latter approach could in the end be more sustainable [4, 51, 52], and is likely to have a greater impact on programme effectiveness and health outcomes in the medium to long term.

There are a number of reasons why CHW programmes need to be adaptable. Firstly, different population groups within a country, and stakeholders within different communities, may have very different health and therefore programme needs, as well as different priorities. Secondly, programmes may need to be adapted for particular local contexts, such as remote areas with poor physical access where operational challenges differ dramatically from more densely populated urban areas. Thirdly, CHW programmes may need to be adapted to local or regional health system arrangements, such the availability of other healthcare providers in the area or the extent of private sector healthcare provision. Additional file 1: Table S3 and the CHW Reference Guide [14] describe a range of questions that need to be considered by policy-makers when deciding whether and how to adapt a programme for different settings or groups.

## Additional considerations

Other issues that may be important to consider in relation to governing CHW programmes at scale include the requirements that scale-up of the programme might impose on the health system (including on managers, healthcare providers, and users) and on other sectors. Also important are factors that might affect the sustainability of the programme, and ways in which national and international stakeholders can be mobilized to support CHW programmes. These issues are discussed further in the chapters on relations with the health system, financing, and planning in the CHW Reference Guide [14] as well as in other papers in this series [53, 54]**.**

Our discussion on governing CHW programmes has several limitations. Firstly, it takes the perspective of policy-makers in formal systems. While this perspective is appropriate for most large-scale CHW programmes, the questions we have outlined may be different when viewing the governing of programmes from a community perspective or the perspective of a small NGO running a local CHW programme, or even a larger NGO managing a substantial CHW programme [55, 56]. The community perspective is addressed in another paper in this series [38]. Secondly, we acknowledge that the diversity of CHW programmes and implementation contexts means that the questions we have outlined may not be appropriate in all cases.

## Areas for future research on governing CHW programmes

While the literature on governing health programmes and systems is growing [9, 11, 57–60], little research has focused on governing CHW programmes specifically [12]. The development and revitalization of large-scale CHW programmes in a number of countries provides an opportunity to explore how different models of governing CHW programmes impact on the quality of care delivered, their responsiveness to local needs, and their sustainability. In Table 2, we outline key areas for future research on governing CHW programmes.

**Table 2:** Areas for future research on governing CHW programmes

• Documenting, through case studies, different models for governing CHW programmes in different settings and how these models work in practice. Important questions include how are these governance arrangements changing over time? How do different stakeholders, including members of communities, view governance in relation to CHW programmes and how do they participate in governing these programmes? To what extent are the arrangements for governing these programmes aligned with national and regional political and health systems goals? Which approaches have worked well, and what lessons can be learned? • What are the impacts for governing CHW programmes of: Scale-up and/or expansion in terms of the range of services offered by CHWs? The professionalization of CHWs, including the formation of representative organizations and the formalization of CHW training? Different models of supporting and incentivizing CHWs? Different models of financing CHW programmes? Digital tools for supporting CHW programmes? Changes in wider national policies that impact on CHW programmes, such as policies on decentralization and on private–public partnerships?
• How can different approaches to governing CHW programmes contribute to mutual accountability of all actors? • Where local participation in governing CHW programmes is not well established, what mechanisms can be used to enhance the accountability of these programmes? • How can the improved governance of CHW programmes lead to more accountable and people-centred health programmes? • How do community and health system governance interact, and how can this interaction be strengthened and aligned?

## Conclusions

The most appropriate and acceptable models for governing CHW programmes depend on communities, on local health systems, and on the political system in which the programme is located. As more and more programmes are scaled up, policy-makers and other stakeholders in each setting need to consider what systems are currently in place and how they might be adapted and aligned with local needs and systems. In this regard, particular attention needs to be paid to community participation and to accountability. Local participation in governing CHW programmes is difficult to achieve at scale, however, without substantial resources, adequate planning, and sustained attention to maintaining local structures. Stakeholders need to consider what resources are needed and how these can be made available.

Where local participation in governance is not well established (for example, because health and political system governance is highly centralized) or is weak, stakeholders need to explore other mechanisms for accountability. Furthermore, it is challenging to include a very local participatory structure for governing a CHW programme within a large-scale programme, and there are few sustained examples of this. For large-scale programmes, formal local governance structures, such as elected local government councils, may need to be relied on.

Governing CHW programmes is complex because of the location of these programmes at the interface between the formal health system and communities—CHW programmes frequently fall outside of the governance structures of the formal health system or are poorly integrated with these. This liminal location, as well as accountability mechanisms that are often weak, increases instability and vulnerability of CHW programmes to health and political system changes. The involvement of a wide range of stakeholders at local, national, and international levels creates additional challenges. All these factors point to the imperative of putting in place appropriate mechanisms for governing CHW programmes. A key weakness of many large-scale programmes is that the questions we have outlined above are not addressed systematically, and mechanisms for governing programmes evolve in an ad hoc way. We hope that the questions we have outlined will provide a useful guide for policy-makers.

## Supplementary Information

**Additional file 1.** Additional tables.

## Data Availability

Data sharing is not applicable to this article as no data sets were generated or analysed during the current study.

## Abbreviations

CHWs: Community health workers
COVID: Coronavirus disease
MOH: Ministry of health
NGO: Nongovernmental organization
PHC: Primary healthcare
SDGs: Sustainable Development Goals
TB: Tuberculosis

## References

[1] World Health Organization (2020). State of the world's nursing 2020: investing in education, jobs and leadership.

[2] Afriyie DO, Nyoni J, Ahmat A (2019). The state of strategic plans for the health workforce in Africa. BMJ Glob Health.

[3] Asamani JA, Akogun OB, Nyoni J, Ahmat A, Nabyonga-Orem J, Tumusiime P (2019). Towards a regional strategy for resolving the human resources for health challenges in Africa. BMJ Glob Health.

[4] Lehmann U, Twum-Danso NAY, Nyoni J (2019). Towards universal health coverage: what are the system requirements for effective large-scale community health worker programmes?. BMJ Glob Health.

[5] Lewin S, Munabi-Babigumira S, Glenton C, Daniels K, Bosch-Capblanch X, van Wyk BE, Odgaard-Jensen J, Johansen M, Aja GN, Zwarenstein M, Scheel IB (2010). Lay health workers in primary and community health care for maternal and child health and the management of infectious diseases. Cochrane Database Syst Rev.

[6] Perry HB, Zulliger R, Rogers MM (2014). Community health workers in low-, middle-, and high-income countries: an overview of their history, recent evolution, and current effectiveness. Annu Rev Public Health.

[7] Bhaumik S, Moola S, Tyagi J, Nambiar D, Kakoti M (2020). Community health workers for pandemic response: a rapid evidence synthesis. BMJ Glob Health.

[8] World Bank (2008). Governance matters 2008: worldwide governance indicators 1996–2007.

[9] Schneider H (2019). The governance of national community health worker programmes in low- and middle-income countries: an empirically based framework of governance principles, purposes and tasks. Int J Health Policy Manag.

[10] Schneider H, Nxumalo N (2017). Leadership and governance of community health worker programmes at scale: a cross case analysis of provincial implementation in South Africa. Int J Equity Health.

[11] Schneider H (2019). The governance of national community health worker programmes in low-and middle-income countries: an empirically based framework of governance principles, purposes and tasks. Int J Health Policy Manag.

[12] Agarwal S, Kirk K, Sripad P, Bellows B, Abuya T, Warren C (2019). Setting the global research agenda for community health systems: literature and consultative review. Hum Resour Health.

[13] Schaaf M, Warthin C, Freedman L, Topp SM (2020). The community health worker as service extender, cultural broker and social change agent: a critical interpretive synthesis of roles, intent and accountability. BMJ Glob Health.

[14] Perry H, Crigler L (Eds.): Developing and strengthening community health worker programs at scale: a reference guide and case studies for program managers and policymakers. USA: USAID Maternal and Child Health Integrated Program (MCHIP); 2014. https://www.mchip.net/sites/default/files/mchipfiles/CHW_ReferenceGuide_sm.pdf. Accessed 1 Mar 2021.

[15] Perry H, Crigler L, Lewin S, Glenton C, LeBan K, Hodgins S (2017). A new resource for developing and strengthening large-scale community health worker programs. Hum Resour Health.

[16] Perry H (Ed.). Health for the People: National Community Health Programs from Afghanistan to Zimbabwe. USA: USAID Maternal and Child Health Integrated Program (MCHIP); 2020. https://pdf.usaid.gov/pdf_docs/PA00WKKN.pdf. Accessed 1 Mar 2021.

[17] Lewin S, Lehmann U: Chapter 4: Governing Large-Scale Community Health Worker Programs. In *Developing and strengthening community health worker programs at scale: a reference guide and case studies for program managers and policymakers.* Edited by Perry H, Crigler L. USA: USAID Maternal and Child Health Integrated Program (MCHIP); 2014. https://www.mchip.net/sites/default/files/mchipfiles/CHW_ReferenceGuide_sm.pdf. Accessed 1 Mar 2021.

[18] World Health Organization (2007). Everybody’s business: strengthening health systems to improve health outcomes—WHO’s Framework for Action.

[19] Baez-Camargo C, Jacobs E: A framework to assess governance of health systems in low income countries. Working paper series No.11. Basel: Basel Institute on Governance; 2011.

[20] Siddiqi S, Masud TI, Nishtar S, Peters DH, Sabri B, Bile KM, Jama MA (2009). Framework for assessing governance of the health system in developing countries: gateway to good governance. Health Policy.

[21] Kooiman J, Bavinck M, Chuenpagdee R, Mahon R, Pullin R (2008). Interactive governance and governability: an introduction. J Transdiscipl Environ Stud.

[22] Meessen B (2020). Health system governance: welcoming the reboot. BMJ Glob Health.

[23] Abimbola S, Negin J, Martiniuk AL, Jan S (2017). Institutional analysis of health system governance. Health Policy Plan.

[24] Bigdeli M, Rouffy B, Lane BD, Schmets G, Soucat A, Bellagio G (2020). Health systems governance: the missing links. BMJ Glob Health.

[25] Pyone T, Smith H, van den Broek N (2017). Frameworks to assess health systems governance: a systematic review. Health Policy Plan.

[26] Wallace C, Farmer J, McCosker A (2018). Community boundary spanners as an addition to the health workforce to reach marginalised people: a scoping review of the literature. Hum Resour Health.

[27] Schneider H, Lehmann U (2016). From community health workers to community health systems: time to widen the horizon?. Health Syst Reform.

[28] LeBan K, Perry H, Crigler L, Colvin C: Chapter 13: Community Participation in Large-Scale Community Health Worker Programs. In *Developing and strengthening community health worker programs at scale: a reference guide and case studies for program managers and policymakers.* Edited by Perry H, Crigler L. USA: USAID Maternal and Child Health Integrated Program (MCHIP); 2014. https://www.mchip.net/sites/default/files/mchipfiles/CHW_ReferenceGuide_sm.pdf. Accessed 1 Mardh 2021.

[29] Strodel RJ, Perry HB (2019). The National Village Health Guide Scheme in India: lessons four decades later for community health worker programs today and tomorrow. Hum Resour Health.

[30] Scott K, Shanker S (2010). Tying their hands? Institutional obstacles to the success of the ASHA community health worker programme in rural north India. AIDS Care.

[31] Lewis M, Pettersson G (2009). Governance in health care delivery: Raising performance Policy Research Working Paper 5074.

[32] Mitchell SM, Shortell SM (2000). The governance and management of effective community health partnerships: a typology for research, policy, and practice. Milbank Q.

[33] Naimoli JF, Perry HB, Townsend JW, Frymus DE, McCaffery JA (2015). Strategic partnering to improve community health worker programming and performance: features of a community-health system integrated approach. Hum Resour Health.

[34] Bossert T (1998). Analyzing the decentralization of health systems in developing countries: decision space, innovation and performance. Soc Sci Med.

[35] McCollum R, Limato R, Otiso L, Theobald S, Taegtmeyer M (2018). Health system governance following devolution: comparing experiences of decentralisation in Kenya and Indonesia. BMJ Glob Health.

[36] Lehmann U, Gilson L (2013). Actor interfaces and practices of power in a community health worker programme: a South African study of unintended policy outcomes. Health Policy Plan.

[37] Strengthening Primary Health Care though Community Health Workers: Closing the $2 Billion Gap**.** https://www.usaid.gov/sites/default/files/documents/1864/USAID_FAH_Report_digital_version_nov21-508.pdf. Accessed 1 Mar 2021.

[38] LeBan K, Perry H. Community Health Workers at the Dawn of a new Era: 9. CHWs’ relationships with the health system and the community. Human Res Health. 2020. 10.1186/s12961-021-00756-4. 10.1186/s12961-021-00756-4 PMC8506091 34641902

[39] Gopinathan U, Lewin S, Glenton C (2014). Implementing large-scale programmes to optimise the health workforce in low- and middle-income settings: a multicountry case study synthesis. Trop Med Int Health.

[40] van Ginneken N, Lewin S, Berridge V (2010). The emergence of community health worker programmes in the late apartheid era in South Africa: an historical analysis. Soc Sci Med.

[41] Victora CG, Aquino EM, do CarmoLeal M, Monteiro CA, Barros FC, Szwarcwald CL (2011). Maternal and child health in Brazil: progress and challenges. Lancet.

[42] Brugha R, Varvasovszky Z (2000). Stakeholder analysis: a review. Health Policy Plan.

[43] Hyder A, Syed S, Puvanachandra P, Bloom G, Sundaram S, Mahmood S, Iqbal M, Hongwen Z, Ravichandran N, Oladepo O (2010). Stakeholder analysis for health research: case studies from low- and middle-income countries. Public Health.

[44] Robert E, Rajan D, Koch K, Weaver A, Porignon D, Ridde V (2020). Policy dialogue as a collaborative tool for multistakeholder health governance: a scoping study. BMJ Glob Health.

[45] Dovlo D, Nabyonga-Orem J, Estrelli Y, Mwisongo A (2016). Policy dialogues - the "bolts and joints" of policy-making: experiences from Cabo Verde, Chad and Mali. BMC Health Serv Res.

[46] Fretheim A, Munabi-Babigumira S, Oxman AD, Lavis JN, Lewin S (2009). SUPPORT tools for evidence-informed policymaking in health 6: Using research evidence to address how an option will be implemented. Health Res Policy Syst.

[47] Lavis JN, Rottingen JA, Bosch-Capblanch X, Atun R, El-Jardali F, Gilson L, Lewin S, Oliver S, Ongolo-Zogo P, Haines A (2012). Guidance for evidence-informed policies about health systems: linking guidance development to policy development. PLoS Med.

[48] Lehmann U, Sanders D (2007). Community health workers: What do we know about them? The state of the evidence on programmes, activities, costs and impact on health outcomes of using community health workers.

[49] Daniels K, Clarke M, Ringsberg KC (2012). Developing lay health worker policy in South Africa: a qualitative study. Health Res Policy Syst.

[50] Hermann K, Van Damme W, Pariyo GW, Schouten E, Assefa Y, Cirera A, Massavon W (2009). Community health workers for ART in sub-Saharan Africa: learning from experience–capitalizing on new opportunities. Hum Resour Health.

[51] Pallas SW, Minhas D, Perez-Escamilla R, Taylor L, Curry L, Bradley EH (2013). Community health workers in low- and middle-income countries: what do we know about scaling up and sustainability?. Am J Public Health.

[52] Rachlis B, Sodhi S, Burciul B, Orbinski J, Cheng AH, Cole D (2013). A taxonomy for community-based care programs focused on HIV/AIDS prevention, treatment, and care in resource-poor settings. Glob Health Action.

[53] Afzal M, Pariyo G, Perry H: Community Health Worker Programs at the Dawn of a New Era: 2. Planning, Coordination, and Partnerships. Human Res Health. 2021. 10.1186/s12961-021-00753-7. 10.1186/s12961-021-00753-7 PMC8506104 34641912

[54] Masis L, Gichaga A, Lu C, Perry H: Community Health Worker Programs at the Dawn of a New Era: 4. Financing. Human Res Health. 2021. 10.1186/s12961-021-00751-9 10.1186/s12961-021-00751-9 PMC8506106 34641893

[55] Haque M (2004). Governance based on partnership with NGOs: implications for development and empowerment in rural Bangladesh. Int Rev Adm Sci.

[56] Schneider H, Nxumalo N (2017). Leadership and governance of community health worker programmes at scale: a cross case analysis of provincial implementation in South Africa. Int J Equity Health.

[57] George A, LeFevre AE, Jacobs T, Kinney M, Buse K, Chopra M, Daelmans B, Haakenstad A, Huicho L, Khosla R (2019). Lenses and levels: the why, what and how of measuring health system drivers of women's, children's and adolescents' health with a governance focus. BMJ Glob Health.

[58] Haley CA, Brault MA, Mwinga K, Desta T, Ngure K, Kennedy SB, Maimbolwa M, Moyo P, Vermund SH, Kipp AM, Team WACSS (2019). Promoting progress in child survival across four African countries: the role of strong health governance and leadership in maternal, neonatal and child health. Health Policy Plan.

[59] Schneider H, Zulu JM, Mathias K, Cloete K, Hurtig AK (2019). The governance of local health systems in the era of Sustainable Development Goals: reflections on collaborative action to address complex health needs in four country contexts. BMJ Glob Health.

[60] Herrera C, Lewin S, Paulsen E, Ciapponi A, Opiyo N, Pantoja T, Rada G, Wiysonge C, Bastías G, Garcia Marti S (2017). Governance arrangements for health systems in low-income countries: an overview of systematic reviews. Cochrane Database Syst Rev.

[61] Lavis JN, Wilson MG, Moat KA, Hammill AC, Boyko JA, Grimshaw JM, Flottorp S (2015). Developing and refining the methods for a 'one-stop shop' for research evidence about health systems. Health Res Policy Syst.

[62] Hall P. The role of interests, institutions, and ideas in the comparative political economy of the industrialized nations. In: Lichbach MI, Zuckerman AS, editors. Comparative politics: Rationality, culture, and structure. Cambridge, UK: Cambridge University Press. 1997.

[63] Lavis JN, Ross SE, Hurley JE, Hohenadel JM, Stoddart GL, Woodward CA, Abelson J (2002). Examining the role of health services research in public policymaking. Milbank Q.

