# Supplementary material for: Community health workers at the dawn of a new era: 3. Programme governance
Source: Health Res Policy Syst. 2021 Oct 12;19(Suppl 3):129. doi: 10.1186/s12961-021-00749-3 (PMC8506073; doi:10.1186/s12961-021-00749-3)
Supplement: Supplementary file 1 — Additional file 1. Additional tables. [file 12961_2021_749_MOESM1_ESM.docx]

**Supplementary Table 1. Health systems governance principles (from [20] p. 13)** ^a^

| **Governance principle** | **Explanation** |
| --- | --- |
| Strategic vision | Leaders have a broad and long-term perspective on health and human development, along with a sense of strategic directions for such development. There is also an understanding of the historical, cultural and social complexities on which that perspective is grounded. |
| Participation and consensus orientation | All men and women should have a voice in decision-making for health, either directly or through legitimate intermediate institutions that represent their interests. Such broad participation is built on freedom of association and speech, as well as on capacities to participate constructively. Good governance of the health system mediates differing interests to reach a broad consensus on what is in the best interests of the group and, where possible, on health policies and procedures. |
| Rule of law | Legal frameworks pertaining to health should be fair and enforced impartially, particularly the laws on human rights related to health. |
| Transparency | Transparency is built on the free flow of information for all health matters. Processes, institutions and information should be directly accessible to those concerned with them, and enough information is provided to understand and monitor health matters. |
| Responsiveness | Institutions and processes should try to serve all stakeholders to ensure that the policies and programs are responsive to the health and non-health needs of its users. |
| Equity and inclusiveness | All men and women should have opportunities to improve or maintain their health and well-being. |
| Effectiveness and efficiency | Processes and institutions should produce results that meet population needs and influence health outcomes while making the best use of resources. |
| Accountability | Decision-makers in government, the private sector and civil society organizations involved in health are accountable to the public, as well as to institutional stakeholders. This accountability differs depending on the organization and whether the decision is internal or external to an organization. |
| Intelligence and information | Intelligence (including data on changes in disease burden and on health systems performance; local evidence on contextual issues; and options for policy making) and information are essential for a good understanding of health systems, without which it is not possible to provide evidence for informed decisions that influence the behavior of different interest groups that support, or at least do not conflict with, the strategic vision for health |
| Ethics | The commonly accepted principles of health care ethics include respect for autonomy, non-maleficence (a principle of bioethics that asserts an obligation not to inflict harm intentionally), beneficence (actions to benefit others) and justice. Health care ethics, which includes ethics in health research, is important to safeguard the interest and the rights of all those served by the health system, including patients. |

*Footnote:* ^a^The reference numbers in this table refer to references in the main paper

**Supplementary Table 2. Cross-country comparison of CHW program governance (based on Appendix 1 in [14] and [16])^a, b^**

|  | | **Key governance considerations** | **Relevance and importance of the issue** | **Country** | | | | |
| --- | --- | --- | --- | --- | --- | --- | --- | --- |
|  |  |  |  | **Brazil**  **Family Health Program** | **Pakistan**  **Lady Health Worker Program** | **India**  **ASHA Program** | **South Africa**  **Ward-based Primary Health Care (PHC) Outreach Teams** | **Ethiopia**  **Health Extension Program** |
| **Inception year (as a national program)** | |  |  | 1994 | 1994 | 2005 | 2011 | 2003 |
| **Types of CHW** | Is there one or are there several types of CHW? | | Historical experiences, both negative and positive, may shape views and responses. Diversity and unclear boundaries can lead to conflict among health workers and/or gaps in provision | Community Health Agent (CHA) | Lady Health Worker (LHW) | Accredited Social Health Activist (ASHA) | Community Health Worker | Health Extension Worker (HEW)  Health Development Army (HDA, formerly called Community Health Promoter, or CHP)  Various other types of CHWs including Community-Based Reproductive Health Agent (CBRHA) and HIV Lay Counselor |
| **Size of the program** | Is this a national or small-scale local program? | | Size and scope of program impacts on the complexity of governing the program | 265,000 working in 43,000 family health care teams | 100,000 | 1.3 million ASHAs have been selected (across 31 States and Union Territories) | This is not clear, but there may be around 72,000 CHWs, attached to various NGOs and programs | 40,000 HEWs; 3 million Women Health Development Army Volunteers in 15,000 *kebeles* (communities) |
| **Historical legacies** | Are there important health system legacies in relation to how programs are governed, and in terms of key players and specific institutions, financial or delivery arrangements that may shape CHW policy-making?  To what extent are these historical legacies in alignment with the planned policy? What scope is there for building on or re-shaping the policy or bypassing these legacies? | | Historical legacies may define, constrain or facilitate CHW policies. Policy may be shaped by previous experience or existing practices. Legacies will determine what actors think of policy and how they will enact and react to it. | The program has its antecedents in a regional program in Ceará State, where it emerged from an emergency response to a severe draught [63]. | In 1993 Pakistan established the Prime Minister’s Program for Family Planning and Primary Health Care that employed CHWs to provide PHC services in their communities. The program subsequently only employed female CHWs. | ASHAs are the most recent incarnation of CHWs in a long history of national and state-level CHW programs in India.  In many states, the ASHA program built upon pre-existing CHW programs, including the national Village Health Guide scheme [29].  The Chhattisgarh Mitanin CHW program, launched in 2003 as a precursor to the ASHA program, has retained the name “Mitanin” for their health workers but has otherwise been encompassed by the ASHA program. | South Africa has never had a large-scale, national CHW program, but has had numerous smaller and larger CHW projects since the 1980s. In the 1990s and early 2000s these CHWs often worked as volunteers and single-purpose workers, with insecure funding. The present emerging national program builds on this “stock” of CHWs and their experience. | In the 1997/8 fiscal year the Ethiopian Federal Ministry of Health launched the National Health Sector Development Program (HSDP). This program shifted the health system’s focus from predominantly curative to more preventive and promotive care and prioritized the needs of the rural inhabitants, who constitute 83% of the Ethiopian population. The “Accelerated Expansion of Primary Health Care Coverage” and the Health Extension Program (HEP) was launched in 2003. |
| **Health system structure** | How does CHW policy fit into wider health governance structures? | | CHW programs in many settings remain peripheral to the rest of the health system. This undermines their legitimacy, hampers alignment of tasks and responsibilities, and may cut them off from mainstream funding sources. | There are three levels of health care provided in Brazil with strong emphasis on PHC. This care is the entry point to more advanced care, but also has promotive and preventive components. Family Health Care Teams are the main service providers and are comprised of one doctor, one nurse, one auxiliary (assistant) nurse, and a minimum of four CHWs. | There are three tiers of governance in the Pakistani public health system: federal, provincial and district. Responsibility for health services rests with provinces, with the exception of a national Ministry of Regulation.  The district level is responsible for allocation and supervision of LHWs. All tiers of government are involved in the LHW program, and LHWs are integral to service delivery of most community health initiatives in the country. | The rural public health system is designed from the village to the state level. In addition to an ASHA worker, each village should have an Anganwadi Worker (AWW). A multipurpose worker (MPW) and an auxiliary nurse midwife (ANM) are employed to conduct outreach to villages on a monthly basis. The MPW works out of the sub-center, a clinic that serves several villages. The ANM is based in the PHC center, a larger clinic that is supposed to be open 24/7 and includes a doctor. Referrals can be made from there to the community health center (CHC) and district hospital. | South Africa introduced a district health system shortly after its first democratic election in 1994. The most recent health sector reforms, aiming at revitalizing PHC, have introduced community health services consisting of clinics, school health teams, specialist teams, and PHC outreach teams at community and household levels. First-level hospital care is rendered through district hospitals, and referrals take place from these to secondary and tertiary hospitals. | The Ethiopian health system is decentralized and has been reorganized into three tiers: (1) PHC Units, each comprised of a health center, five satellite health posts, and a district/*woreda* hospital; (2) zonal/general hospitals; and (3) specialized/referral hospitals. |
| **Structure of the program** | How is the program integrated or  aligned with the formal health system? | | Signals how the program is located in the governance structures of health system. | CHAs operate as members of the family health care teams (*Equipo de Saúde Familiar*) that are managed by municipalities. These teams are based within the Family Health Program clinics and provide services to 600-1,000 families or a maximum of 4,500 people. | LHWs are attached to a local health facility, but they are primarily community-based, working from their homes. The homes of LHWs are named Health Houses, and emergency treatment and care are provided from these houses. | ASHAs are based in their villages but refer people to their local CHC and PHC center. Village Health and Sanitation Committees (VHSCs), composed of village residents including the ASHA, also provide support for the ASHA’s activities.  Although service delivery varies by state, in general, ASHAs are expected to attend weekly meetings at their local PHC center and make home visits in the community as needed. They work approximately 2 hours a day, four days per week. | The new system for the first time sees CHW as part of the system of service delivery. Similar to the Brazilian model, PHC outreach teams consist of 5-6 CHWs supervised by a nurse. They render services in households and communities, and refer patients to clinics as needed. | The aim of the HEP is to “provide equitable access to promotive, preventive and select curative health interventions through 40,000 government-salaried Health Extension Workers (HEWs), two per *kebele* (neighborhood), located at a health post. The HEWs, young local women with at least a grade- 10 education, are recruited by *Kebele* and *Woreda* Councils and given one year of training prior to employment with the *Woreda* Health Office. |
| **Employment status of CHWs** | Are CHWs employees of the state and/or appointed by communities? | | Signals who CHWs are accountable to, and how firmly embedded they are in structures of the health system. | State employees | State employees | Considered volunteers but receive a government stipend (without benefits). | Employed by NGOs who in turn have service contracts with state health services at district level. | State employees |
| **Program financing** | How are CHW programs financed? | | How CHW programs are financed reflects both national and local priorities and is also a key governance mechanism. | The Family Health Program is co-funded by states and municipalities, but regulated by the national government. The CHW program is an integral part of Family Health Program and thus funded as part of it. | The Pakistani government is the largest funder of LHW services, although the program has been underfunded since its inception. The vast majority (around 70%) of the costs are comprised of LHW stipends, drugs and contraceptives. 4% of overall costs are for training. | In 2006, the Ministry of Health and Family Welfare (MoHFW) stipulated that the program would cost US$185 per ASHA per year. This included the costs of selection, social mobilization, training, drug kits, identity cards and support for ASHAs through the PHCs and supervisors. This estimate did not include the ASHAs’ stipends, which were to come from the budgets of other MoHFW initiatives. | In the past, programs were largely funded from external grants. The new program will increasingly be funded through the health budget. | Financed by a mix of national and sub-national government entities, bilateral and multilateral donors, and NGOs.  At the local level, financing and planning are decentralized and the *woredas* receive block grants to cover HEP expenses. |
| **Program scale-up** | Will the program be taken to scale and, if so, how will this occur? | | CHW programs generally aim to improve access to and quality of health care for remote and poor communities. | In 1990 there were 78,805 CHAs and there are now over 236,000 CHAs that provide services to 98 million people (out of a total population of 210 million) within 85% of Brazil’s municipalities. | A 2000 evaluation estimated that 150,000 LHWs were needed to obtain optimal coverage in the country. Since then there has been a consistent scale-up, to 90,074 in 2008 and 100,000 at present. This increased LHW coverage is in more rural and poorer areas, but the program still does not reach the most disadvantaged areas. | Initially (2005-2008) the ASHA program was a component of the National Rural Health Mission only in 18 “High Focus States” and in the tribal districts of other states. In 2009 the program was extended to cover the entire country. At present there are now 1.3 million ASHAs. | The intention is to roll the program out nationally. Numerous pilot sites are operational at this stage and are being carefully monitored and evaluated. | There have been four HSDPs since its inception in 1997. Rollout has occurred in a step-wise manner, in which the speed was influenced by available resources for health posts and presence of eligible women to become HEWs. |
| **Local (community) governance** | How are communities involved in decision-making about CHW activities at local level? Are they involved in selection? Can they hold CHWs to account? Can they influence decision-making about funding, support, etc.? | | Community acceptance and therefore community participation is considered central to any CHW program, but mechanisms of community participation in governing programs are often poorly developed and dysfunctional. | Community governance functions through national, state and municipal health councils, with more than 5,500 municipal councils participating. Councils are comprised of 50% users, 25% health workers and 25% health managers and service providers. Health conferences are also held every four years to propose directives for health policies. | The selection committee for LHWs includes a person nominated by the local community, and potential LHWs are identified through local community structures where possible. Program planning, implementation, and monitoring and evaluation also should include community participation. However, the extent to which this occurs varies. | ASHAs are to be selected by and accountable to the local village level government, called the Gram Panchayat, through a participatory process involving the whole village. After selection, ASHAs are to work closely with the Village Health and Sanitation Committee (VHSC). This committee is comprised of key stakeholders in the village. | All health districts have district health councils who have representation from civil society. Implementation is at an early stage and uneven throughout the country. Furthermore, community health committees are supposed to oversee the functioning of service delivery in communities and facilities. | There are active health committees involved in the selection and oversight of HEWs and they are involved in these activities with CHPs in some geographical areas. Additionally, the *kebele* council is supposed to be involved in every step of the HEP from program planning through to evaluation. |
| **Relationship with the formal health services** | What are lines of reporting and accountability? What is the level of integration? | | In many CHW programs, links with the formal health services are tentative and not well thought through. Professionals at the first formal level of service delivery (e.g., health centers) often resist engagement with and support for CHWs. | CHAs are managed by local nurses who spend half their time working in the local clinic. Thus, CHAs are closely integrated into formal health services. They also have strong referral systems in which they report any ill person within their catchment area to a nurse. | All LHWs are attached to a First- Level Health Facility in the form of either a rural health center or a basic health unit. LHWs generally receive their supplies from these facilities, although there are challenges with insufficient staff and stock outs at local clinics. | Although ASHAs are supposed to be representatives of and accountable to the people, they receive their payments through the ANM at the PHC and are often treated as extensions of the health system. | CHWs are managed by nurses and structurally linked to the formal health services. Prior practices and experiences were very mixed and dependent on links between NGOs and health services. They were often dependent on personal relationships as well. | HEWs are full members of the formal health workforce. They staff health posts and are responsible for the Women’s Development Army Volunteers and model families. Many HEWs work in hard-to-reach and isolated areas, where supervision, supplies and referrals remain a challenge. |

*Footnote:* ^a^ The reference numbers in this table refer to references in the main paper. ^b^ The recently released compendium of case studies of national CHW programs has a complete description of these five programs [16].

*Abbreviations*: ASHA: Accredited Social Health Activist; CBRHA: Community-Based Reproductive Health Agent; CHA: Community Health Agent; CHC: Community Health Center; CHP: Community Health Promoter; CHW: Community Health Worker; HEP: Health Extension Program; HEW: Health Extension Worker; HSDP: Health Sector Development Program; LHW: Lady Health Worker; NGO: non-governmental health organization; PHC: primary health care; VHSC: Village Health and Sanitation Committee.

**Supplementary Table 3. Governance structures and mechanisms in relation to the definition, selection, training, support and remuneration of individual CHWs (based on**

**Appendix 1 in [14])^a^**

| **Governance issue** | **Brazil** | **Pakistan** | **India** | **South Africa** | **Ethiopia** |
| --- | --- | --- | --- | --- | --- |
| **CHW criteria** | CHAs work in the community where they are from or are permanently residing. The only other selection criterion is completion of primary school. | LHWs are females who have a minimum of eight years of education. They also must be between 18 and 45-50 years old, reside in and be acceptable to or recommended by their community, and preferably be married with children. | ASHAs have a class-eight education or higher and preferably are between the ages of 25 and 45 years of age. ASHAs are required to be “daughters-in-law” of the village, i.e., married women (or widowed or divorced) so that they are likely to live in the village for the foreseeable future. | Criteria for selection vary, but in most cases, CHWs who were active through NGOs prior to the introduction of a national program are being drawn on to continue rendering services. | HEWs are adult females who have completed 10^th^ grade. HEWs are supposed to work in or close to their native community/permanent residence. |
| **Selection process** | CHAs are hired by their municipalities based on their demonstrated abilities while addressing simulated community problems during the selection process. | LHW are selected using a clearly delineated process. LHW posts are advertised and applicants are then interviewed and selected based on pre-set criteria by a selection committee. | Local governance structures and the wider community should be involved in ASHA selection. However, these selection processes are not always adhered to. | Selection processes vary widely, depending on the NGOs who contract with the CHWs. | Health committees are involved in the selection of HEWs from the local community.  WDA Volunteers are either nominated and elected by the community or they are selected by HEWs and approved by the community. |
| **Scope of work** | One of the goals of the Family Health Program is to promote community engagement and to analyze the community’s needs. Thus, CHAs are expected to serve as the link between the Family Health Care Teams and the surrounding community.  Family Health Care Teams provide comprehensive care through promotive preventive, recuperative, and rehabilitative services.  Services provided by CHAs include the promotion of breastfeeding, the provision of prenatal, neonatal and child care, the provision of immunizations, and the clinical management of infectious diseases, including screening for and providing treatment for HIV/AIDS and tuberculosis, support for persons with non-communicable disease, and assistance with the management of those who have mental and social problems. CHAs register the households where they work and are expected to empower their communities and link them to the formal health system. | LHWs are expected to link the community to formal health services and to be members of the community where they work. They also provide a range of community development services and participate in community meetings.  The LHW program has evolved over time. LHWs’ scope of services has grown from an initial focus on mostly maternal and child health; it now also includes participation in large health campaigns, newborn care, community management of tuberculosis, and health education on HIV/AIDS. | The government of India describes the ASHA’s role as having three key components. First, ASHAs are to play an important role in achieving national health and population policy goals. Second, they are to link rural people with the health system. Third, they are to serve as social change agents who will create awareness on health and its social determinants and mobilize the community towards local health planning and increased utilization and accountability of the existing health services.  Anganwadi Workers (AWWs) provide basic child health information, medicine and nutritional supplementation to children younger than 6 years of age, pregnant and lactating women, and adolescent girls. | A PHC outreach team will initially be responsible for:  • Identifying and capturing details of people who live in the households in the catchment area and assessing those who are most at risk;  • Providing health promotion and prevention;  • Testing for HIV and screening for TB;  • Checking immunization status of children;   - Facilitating use of antenatal care early in pregnancy and use of contraception; and - Responding to the local burden of disease. | HEWs are full-time employees who are supposed to split their time between health posts and the community. HEWs should spend at least 80% of their time in these community-based activities, although considerable anecdotal evidence suggests this is not the case.  HEWs’ main role is in health promotion, disease prevention, and treatment of uncomplicated and non-severe cases of malaria, pneumonia, diarrhea, malnutrition and measles in the community. HEWs provide a range of services including: prevention/health promotion/health education; community-based distribution of health-related commodities; ongoing care or support for people with a chronic illness (e.g., HIV/AIDS); and participation or support role in campaign-type activities. They also provide immunizations, injectable contraceptives, basic first aid, as well as diagnosis and treatment of pneumonia, diarrhea and malaria. |
| **Training** | The national Ministry of Health –with Ministry of Education approval – is responsible for the training of CHAs in Brazil and trains them in regional health schools. CHAs receive eight weeks of training from local nurses, followed by four weeks of supervised field work. This includes training on home visits, how to conduct a family census, and then on specific priority health care interventions. CHAs receive monthly and quarterly ongoing education training during meetings. CHAs are also trained by nurses and state health secretariat staff in their local clinics; these trainers undergo an 80-hour training module. | LHWs are trained for three months on PHC in classrooms and then have one year of on-the-job training. This should include one week of training per month for a period of 12 months and 15 days of refresher training each year thereafter, although there is substantial variation in training patterns across provinces. The Federal Project Implementation Unit is responsible for approval of all LHW training and, with the Ministry of Health, develops the training curriculum, organizes and coordinates training, and trains master trainers while Provincial and District Project Implementation Units are responsible for the local trainings. | ASHAs are to receive 23 days of training over their first year, based on five training manuals. They are then to receive 12 additional days of training each year thereafter. Two additional training modules have just been added to the training regimen. ASHA training has in some states been outsourced to NGOs, and in other states is being conducted by health professionals within the public system. Training generally takes place in a cascading manner, by which state teams are trained and then pass on their training knowledge to district training teams. These district teams then pass on their training to block-level ASHA trainers. ASHAs are then be trained at the block or sub-block level. | The training existing CHWs have received varies widely, and has been provided by a wide range of NGOs and training providers. The MOH is now aiming to standardize training, although this process is still awaiting finalization. | HEWs have one year of pre-service training conducted by trainers who are capacitated using a train-the-trainer approach. HEW training is a collaboration of the Ministry of Health and the Ministry of Education and occurs at 40 Technical and Vocational Education Training Schools.  WDA Volunteers have a brief initial training that is conducted by the HEWs that is less than 3 weeks in length.  These women come from model families and are given 96 hours of training on prevention of communicable diseases, family health, environmental and household sanitation, and health education. |
| **Feedback and supervision** | CHAs are supervised by nurses and physicians from the local health centers. Supervisory nurses spend 50% of their time in these supervisory roles and the rest of their time staffing the local health center, a factor that has been identified as a critical component to the program’s success. | Supervision is highly organized and tiered in the Pakistani LHW program. LHWs are each attached to a public health clinic and are supervised on a monthly basis by an LHW supervisor (LHS). There are two layers of supervision above the LHS. LHWs should have community-based supervision at least once a month in which supervisors meet with clients and with the LHWs in the community where the LHW works, review the LHW’s work, and jointly make a work plan for the next month. | According to national guidelines, there is to be one ASHA Facilitator for every 20 ASHAs. The Facilitator is to help with the selection of the ASHA, run monthly ASHA meetings, establish a system to respond to ASHA grievances, accompany ASHAs on home visits, maintain records of ASHA activities, attend Village Health and Nutrition Days with the ASHAs, and attend monthly Block PHC meetings. The ASHA Facilitator is supervised at the Block level by the Block Community Mobiliser, who is in turn supervised by the District Mobilization/  Coordination Unit, which liaises with the state-level ASHA resource center. | Feedback and supervision is presently provided through NGOs but will in future be provided through the nurse supervisor attached to every outreach team. | HEW supervision appears to vary across the history of the program and geographical contexts. In 2005 HEWs had relatively high levels of supervision with an average of three supervisory visits over the course of nine months. There are supposed to be multiple levels of HEW supervision, including the *woreda* supervisory team that is comprised of a health officer, public health nurse, environmental/hygiene expert, and a health education expert.  HEWs supervise other health workers such as WDA Volunteers and traditional birth attendants. |
| **Compensation/**  **incentives** | CHAs are salaried, full-time workers, but there is a large variation throughout the country in their salary. CHAs are supposed to earn at least the national minimum wage of ~US$ 112 each month. | LHWs receive a salary of about US$ 343 per year and are not supposed to engage in any other paid activity, although some do. The LHW stipend is often the only source of family income and is a critical family support. | Although ASHAs are considered volunteers, they receive outcome-based remuneration for facilitating institutional deliveries, immunization, family planning (surgical sterilization) and toilet construction. More recently, an incentive of US$ 4.60 (Rs.250) has been established for providing home-based newborn care. Facilitating institutional deliveries is the most common activity for which ASHAs receive payments. ASHAs are also compensated for training days, attending meetings, and additional health-related activities. The amounts vary from state to state. | In most provinces in South Africa, NGOs receive funding from the MOH to contract with and pay CHWs. More recently, at least one province has decided to contract with CHWs directly and put them onto the government payroll. Salaries are approximately at the national minimum wage. | HEWs are regular employees with a regular salary of US$ 84 and benefits. A range of non-financial incentives have been effective with WDA Volunteers, including formal recognition, ongoing mentorship, certification and community celebrations. |
| **Career opportunities** | No structured opportunities for career advancement for CHAs exists. However, many move to higher-level health-related positions after obtaining additional training. | The LHW Program offers professional advancement opportunities for LHWs. LHWs can receive additional training to serve as an LHS, which is an incentive for good performance. | Career advancement within the program for ASHAs is limited. | The issue of career development is not addressed in the new policy, but in several provinces pilots are underway to provide career paths into professions such as nursing and social work. | HEWs who enroll in additional training can qualify as registered nurses. |

*Footnote:* ^a^ The reference numbers in this table refer to references in the main paper

*Abbreviations*: ASHA: Accredited Social Health Activist; AWW: Anganwadi Worker; CHA: Community Health Agent; CHW: Community Health Worker; HEW: Health Extension Worker; LHS: Lady Health Supervisor; LHW: Lady Health Worker; MOH: ministry of health; NGO: Non-governmental health organization; PHC: Primary health care; WDA: Women’s Development Army
